# Experimental Investigations on the Pull-Out Behavior of Tire Strips Reinforced Sands

**DOI:** 10.3390/ma10070707

**Published:** 2017-06-27

**Authors:** Li-Hua Li, Yan-Jun Chen, Pedro Miguel Vaz Ferreira, Yong Liu, Heng-Lin Xiao

**Affiliations:** 1 School of Civil Engineering, Architecture and Environment, Hubei University of Technology, Wuhan 430068, China; lilihua466@163.com (L.-H.L.); 931031cyj@sina.cn (Y.-J.C.); xiao-henglin@163.com (H.-L.X.); 2 State Key Laboratory of Geomechanics and Geotechnical Engineering, Institute of Rock and Soil Mechanics, Chinese Academy of Sciences, Wuhan 430071, China; 3 Department of Civil, Environmental and Geomatic Engineering, University College London-UCL, London WC1E 6BT, UK; 4 Department of Civil & Environmental Engineering, National University of Singapore, Engineering Drive, Singapore 119576, Singapore; ceeliuy@nus.edu.sg

**Keywords:** waste tires, reinforced soil, pull-out tests, geogrid, load displacement behavior, frictional resistance, interface properties, rubber

## Abstract

Waste tires have excellent mechanical performance and have been used as reinforcing material in geotechnical engineering; however, their interface properties are poorly understood. To further our knowledge, this paper examines the pull-out characteristics of waste tire strips in a compacted sand, together with uniaxial and biaxial geogrids also tested under the same conditions. The analysis of the results shows that the interlocking effect and pull-out resistance between the tire strip and the sand is very strong and significantly higher than that of the geogrids. In the early stages of the pull-out test, the resistance is mainly provided by the front portion of the embedded tire strips, as the pull-out test continues, more and more of the areas towards the end of the tire strips are mobilized, showing a progressive failure mechanism. The deformations are proportional to the frictional resistance between the tire-sand interface, and increase as the normal stresses increase. Tire strips of different wear intensities were tested and presented different pull-out resistances; however, the pull-out resistance mobilization patterns were generally similar. The pull-out resistance values obtained show that rubber reinforcement can provide much higher pull-out forces than the geogrid reinforcements tested here, showing that waste tires are an excellent alternative as a reinforcing system, regardless of the environmental advantages.

## 1. Introduction

The number of waste tires has rapidly increased in the recent decade. Statistical data [1] has shown that the European Community generated an estimated 4.5 million tons of new tires in 2010, while 289 million tires were replaced that year; other countries have displayed similar data (according to [2,3,4,5,6]). Thus, waste tire disposal has become a serious environmental problem in many countries. Researchers have proposed several beneficial uses for waste tires, particularly since waste tires have the potential to be used as construction and building materials (due to their high strength and durability). Tests performed on a solid tire, produced in the 1920s and aged naturally in the woods for more than 80 years, have shown that whilst a crust had formed on the surface, the material located a few millimeters from the surface maintained the same properties as the natural rubber [7]. It is possible to infer that with the new additives used nowadays, tires are likely to have a higher durability, particularly if protected from contact with air and light.

In geotechnical engineering, artificial fibers have been successfully used to enhance the properties of different types of soils. Sands reinforced with polypropylene fibers have shown better strength and stiffness [8,9], and have also shown that the reinforcement is still active, even at stresses on the 3 to 5 MPa range [8]. The addition of fibers has also improved the mechanical properties of a residual soil from Hong Kong, even when different sample preparation procedures are used [10]. Particularly important is the use of tire fibers (tire shreds) used to reinforce clayey soil, which show that a similar improvement in the mechanical properties can be obtained with these types of fibers [11]. Shredded tires mixed with sands and other soils have also been used as lightweight fills, which are now widely used in embankment fills, retaining walls, and bridge abutment backfills (e.g., [1,12,13,14,15,16,17]).

In addition, to help reduce environmental pollution, tire-reinforced soils also have other advantages, such as low cost and simple construction processes. The authors of [18] have shown the benefits of reinforcing dikes and embankments with whole tires, whilst [19,20] found that with the addition of tire shreds, the shear strength properties of the reinforced soil were higher than the properties of the unreinforced soil. If waste tire particles are added to a clayey soil and mixed with cement, the performance of the cement admixed soil can be significantly improved [21]. They carried out a series of field-scale pull-out tests to investigate the pull-out behavior of cell-type waste tires in reinforced soil. The authors observed that the ultimate pull-out resistance of cell-type tires is around 1.25 times higher than that of commercially available geocells. By conducting laboratory plate load tests, [6,22] verified that the bearing capacity of foundations can be significantly improved by adding treads and sidewalls of waste tires in the soil.

Although tire-reinforced sands are currently being used in practice, there are only a small number of studies that focus on the load-deformation behavior of this kind of reinforcement material, particularly through laboratory pull-out tests. Therefore, this study is aimed at addressing the lack of data with regard to pull-out tests of tires, by testing waste tires cut into strips (30 mm in width and 15 mm in thickness) that serve as a reinforcing material in sands. This was also done to reduce the energy required for the shredding process. In these tests, the relative displacement at the interface was monitored throughout the pull-out process to investigate the pull-out behavior and the load-deformation characteristics of the tire-reinforced sands. The results are also compared to pull-out tests performed in uniaxial and biaxial geogrids, under the same conditions and vertical stresses.

## 2. Laboratory Pull-Out Tests

### 2.1. Test Apparatus

**Figure 1:**
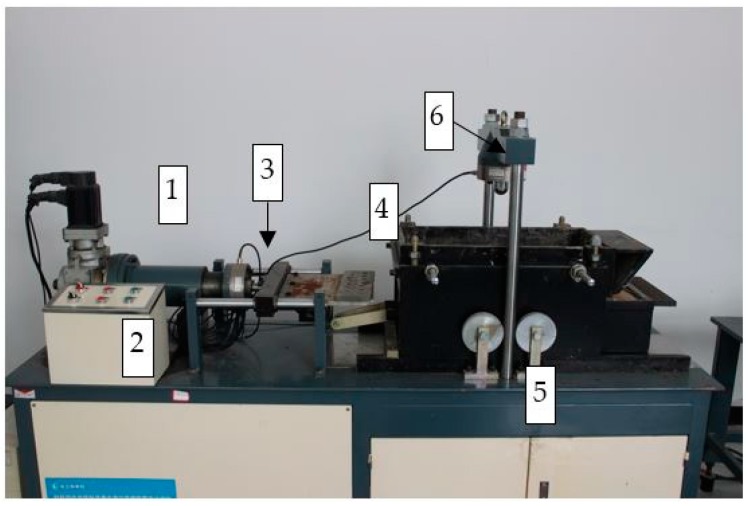
Pull-out apparatus: (1) horizontal loading system; (2) control panel; (3) horizontal load cell; (4) pull-out clamp; (5) vertical loading system; (6) vertical load cell.

The pull-out tests were conducted on a TGH-3C-Geosynthetics pull-out test apparatus, as shown in Figure 1. The apparatus was developed by the Yangtze River Scientific Research Institute, China.

The equipment has a large shear box with inner dimensions of 600 mm in length, 300 mm in width, and 150 mm in height. Once the sample is set up, the upper and lower parts of the shear boxes can be fixed. Test data was automatically recorded using load cells and displacement gauges, using a purposely designed data acquisition software. The horizontal and vertical loads were applied using a strain-control system.

### 2.2. Materials

**Figure 2:**
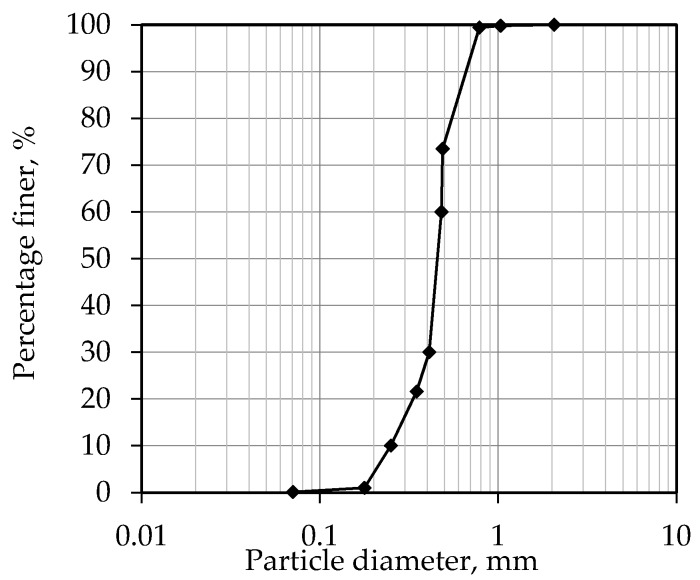
Particle size distribution of Fujian Standard sand.

Table 1 shows the physical properties of the sand samples. This particular type of sand is referred to as *Fujian Standard Sand* in China. Figure 2 shows the particle size distribution of the sand. During the tests, the sand samples were kept dry and clean. The uniaxial and biaxial geogrids were produced by Tensar International. The mechanical properties of these two types of materials (i.e., the tire strips and geogrids) were determined by direct tensile tests; Table 2 and Table 3 contain a summary of the test results. Table 3 has four different types of tire strips, representing different tire wear levels seen on waste tires (Type 3 has the highest tire wear level); Type 4 has the lowest tire wear level; and finally, Types 1 and 2 have a similar and intermediate tire wear level.

### 2.3. Test Methods

**Figure 3:**
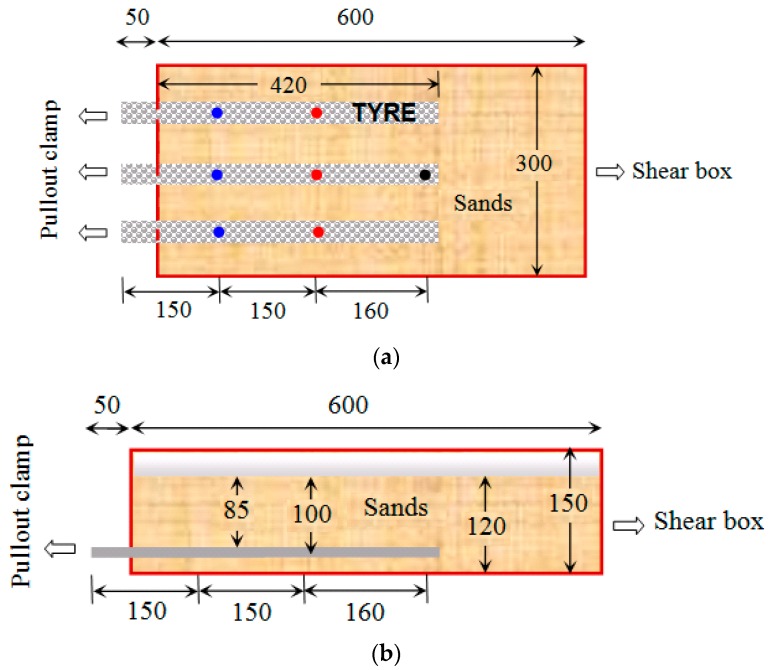
Layout of the tire strips on the shear box: (**a**) plan view; (**b**) elevation. All dimensions in mm.

In the pull-out box, three similar tire strips were laid on a 20 mm thick layer of compacted sand. The compaction was controlled by the relative sand density. Sand was then compacted, above the tire strips, in layers of 20 mm until a height of 120 mm was achieved. The ends of the tire strips located outside the box were attached to the pull-out clamp, as shown in Figure 3. The length of strips inside the shear box was equal to 420 mm. The relative sand density achieved during sample preparation was 0.95, with a dry density of 1.611 g/cm^3^. The tests using uniaxial and biaxial geogrids followed the same procedure, but the width of the geogrid tested was different. The uniaxial geogrid that was tested had six ribs and a width of 90 mm, while the bi-axial geogrid that was tested had 120 mm in width.

To understand the shearing behavior of the tire strips, seven monitoring points on the three tire strips (represented by the colored dots in Figure 3a) were instrumented with stainless steel piano wires to measure displacements. The three tire strips had the same wear intensity in each experiment, and the displacements recorded at the same distance from the pullout clamp were averaged and denoted as v1, v2, and v3 (e.g., v1 denotes the average of the displacement measured at the three blue points and v2 the average of the displacements measured at the red points on Figure 3a). It is worth mentioning that the pull-out tests of the tire strips and uniaxial and biaxial geogrids were carried out under different normal stresses. The lengths of the three reinforcing materials tested (i.e., tire strips, uniaxial, and biaxial geogrids) were the same (420 mm), and their cross-sections were also ensured to be roughly the same.

Previous trials showed that the pulling speed had a significant influence on the experimental results: the greater the pulling speed, the more obvious the viscous damping characteristics. Therefore, the pulling speed used in the tests was kept constant at 1.0 mm/min, following the value used by [23,24] for pull-out tests on geogrids and cellular reinforcements. After the samples were prepared, normal stress was applied, at an incremental rate of 30 kPa/min, until the test vertical stress was achieved. The vertical stress was than maintained constant for the rest of the test duration. Two minutes after reaching the test vertical stress, a pretension of 0.2 kN was applied by the pull-out clamp on the tire strips. This load was kept constant for an extra two minutes before the start of the test. The test started by applying the pulling speed (mentioned above) and recording the pull-out force, clamp displacement, normal stress, and linear displacements on the rubber strips.

When the pull-out force reached a stable or a peak value, the pulling system continued to pull the reinforcement for an additional 15 mm of displacement before terminating the test—unless damage on any of the elements occurred. Two parallel tests were conducted to ensure reliability and repeatability.

## 3. Experimental Results and Discussions

### 3.1. Pull-Out Load-Displacement Relationship

**Figure 4:**
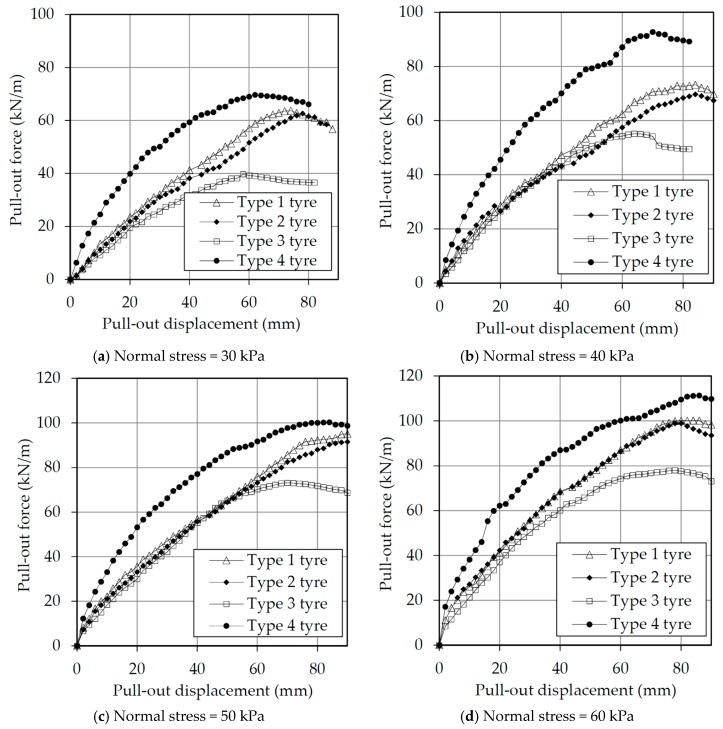
Pull-out load-displacement curves under various normal stresses: (**a**) 30 kPa normal stress; (**b**) 40 kPa normal stress; (**c**) 50 kPa normal stress; (**d**) 60 kPa normal stress.

Figure 4 shows the normalized load-displacement curves obtained from the pull-out tests on the different types of tire strips embedded in sand. The data was normalized by the width of the box for the tire strip reinforcement, as it is assumed that this set up would be repeated in a field application. Figure 4 shows that the peak force increased as the normal stress increased, for all types of tire strips. It is worth pointing out that the Type 4 tire strips showed the highest stiffness when compared to the other types. Also, the differences in stiffness (seen for the other three types of tire strips) are very small and tend to decrease as the normal stresses increase. Once the pull-out force reached a peak, strain-softening characteristics were observed in all tests, indicating a brittle failure mode (as expected).

**Figure 5:**
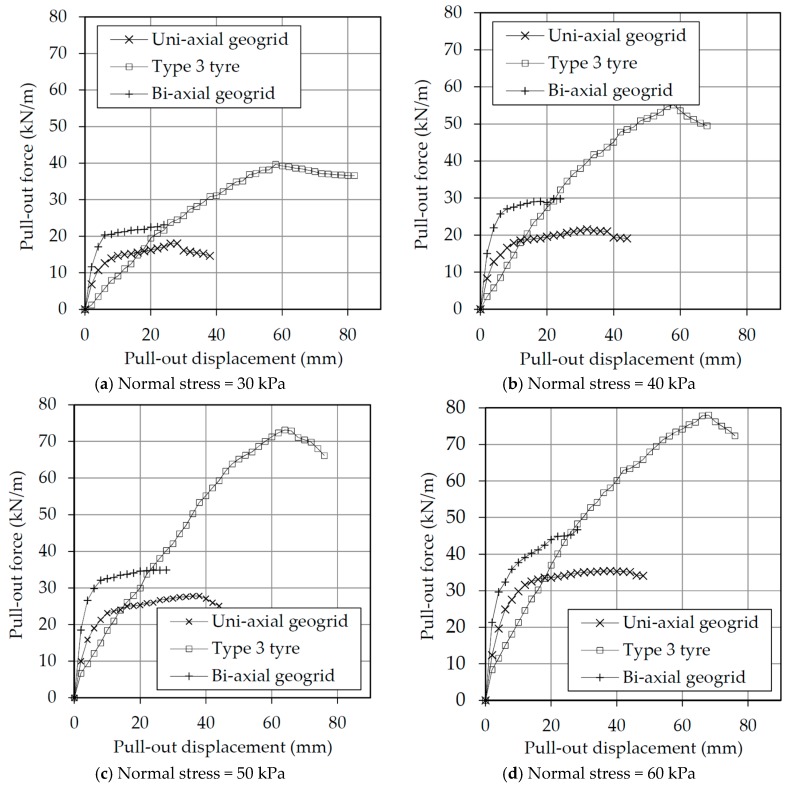
Comparisons between sands reinforced by tire strips and geogrids: (**a**) 30 kPa normal stress; (**b**) 40 kPa normal stress; (**c**) 50 kPa normal stress; (**d**) 60 kPa normal stress.

The pull-out results from the Type 3 tire strips (highest level of tire wear) were compared to the uniaxial and biaxial geogrid test results in Figure 5. Similar to what was seen for the tire strips, the peak values of the pull-out tests on geogrids showed that higher confining stresses yield higher pull-out forces. Whilst the uniaxial and biaxial geogrid have a similar behavior, the biaxial geogrid has always achieved a higher load in all tests and, consequently, a higher stiffness than the uniaxial geogrid.

The pull-out force applied on the geogrids reached an almost stable value at around 10 mm of displacement; thereafter, the increase seen in the pull-out force was very small. The test results also show that the pull-out force of the uniaxial and biaxial geogrids increase much faster than that of the tire strips during the first 10 mm of displacement. In contrast, the load-displacement curve of the tire strips suggests an initial constant and steeper growth rate (lower than the rate found for the geogrids) until the first 20 mm of displacement. This is followed by a slightly slower rate until the force reached a peak value. The tests also show that the uniaxial and biaxial geogrids reach a stable or a limit pull-out load around 20 to 30 mm of displacement, whilst the peak pull-out load (for the tire strips) is achieved at 60 mm of displacement or more. It is important to mention that for displacements around 30 mm, the force achieved by the tire strips are of similar magnitude to the force achieved by the biaxial geogrid for all vertical stresses.

### 3.2. Reinforcement Index

**Figure 6:**
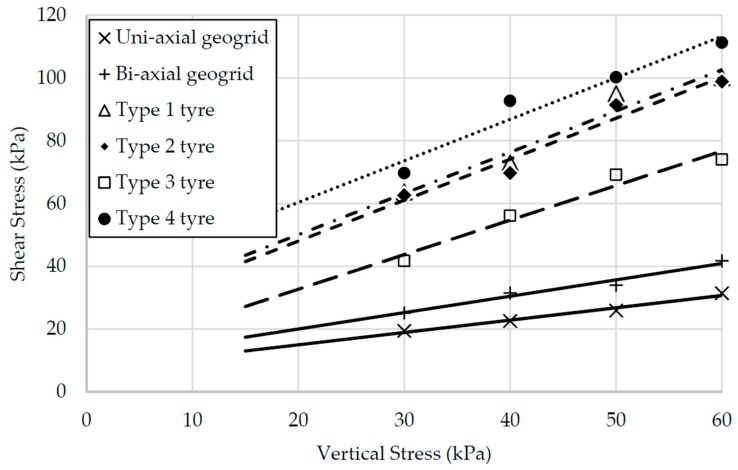
Relationships between normal stress and shear stress.

Figure 6 shows the relationships between the normal stress applied during the tests and the average shear stress mobilized at peak load. The shear stress plotted was calculated assuming that the contact area between reinforcement and soil does not change and there is no progressive failure. This simplification does not take into account other types of failure mode, which are particularly important in the case of the geogrids (with their complex shape) and should yield higher values of stress for all cases tested. The results show that the values obtained for the tire strips plot above the values obtained for the geogrid, showing that the contact interface between the tire strips is capable of mobilizing much larger shear stresses than that of the geogrids.

The Mohr-Coulomb strength parameters, calculated from the pairs of stresses plotted in Figure 6, are shown in Table 4. The results show that even when overestimated shear stresses where used, the shear strength parameters determined for the uniaxial and biaxial geogrids are below those of the pure sand used in the experiments, indicating that the shearing is likely to occur at the interface between the geogrid and the sand. An interesting observation, however, is the apparent shear strength parameters calculated for the rubber interface, some of them reaching values of friction angle above 50°. These values are far higher than the shear strength parameters of the sand itself, indicating that there is some form of interlocking mechanism acting between the tire strips and the sand particles. This mechanism moves the shearing interface into the sand, away from the tire strip-sand interface, allowing the mobilization of higher pull-out loads. Figure 6 and Table 4 also show the differences between the tire types studied, where the major difference in the apparent envelopes is the values of cohesion determined. Therefore, the tire wear level, whilst allowing the mobilization of higher loads, has a much larger influence in the apparent cohesion intercept than in the apparent friction angle.

### 3.3. Tensile Strain

**Figure 7:**
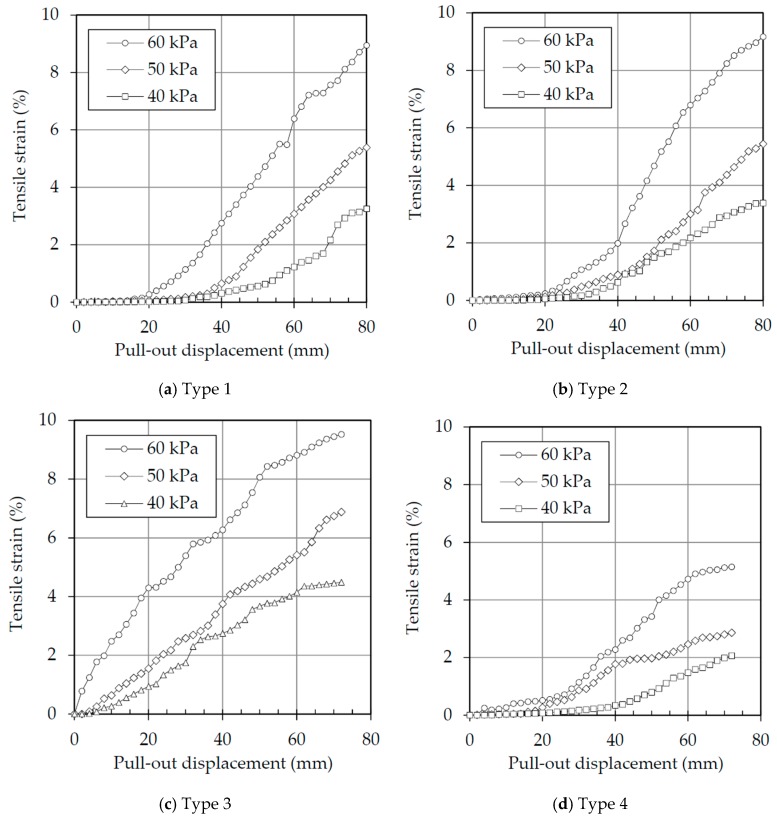
Tensile behavior of tire strips under various wear intensities: (**a**) Type 1 tire strips; (**b**) Type 2 tire strips; (**c**) Type 3 tire strips; (**d**) Type 4 tire strips.

The average tensile strain acting on the end portion of the tire strips was calculated using the difference between the displacement measurements on v2 and v3 (Figure 3a), and was plotted against the pull-out displacement (Figure 7) for all four types of tire strips and three of the normal stresses that were tested. Figure 7a,b shows that the tensile strain curves of Types 1 and 2 tire strips are fairly similar, due to the same tire wear level (as was expected). The figure also shows that at the beginning of the tests with 40 kPa of vertical stress, there is no mobilization of strains until the pull-out displacement reaches 20 mm, indicating that the pull-out force is a result of the strength mobilized by the initial portion of the tire strips only. As the confining stresses increase, the strength mobilization also increases (proportionally to the vertical stress applied).

The only exception to the pattern seen is the Type 3 tire strips (highest tire wear level), where the mobilization of strains starts at the beginning of the test, and is therefore much earlier than the other types.

The level of strains mobilized by the different types of tire strips is coherent with the stiffness values measured by the direct tensile tests (Table 3). Type 4 has the highest stiffness and mobilized the lowest strains, whilst Types 1 and 2 mobilized less strains than Type 3 (given its intermediate stiffness). Type 3 has the lowest stiffness and, therefore, mobilized the larger strains. An important observation is that the strain levels achieved, however, are much lower than the values measured at yield on the direct tensile tests (where strains above 64% where measured for Type 4 and strains higher than 75% for the other three types).

**Figure 8:**
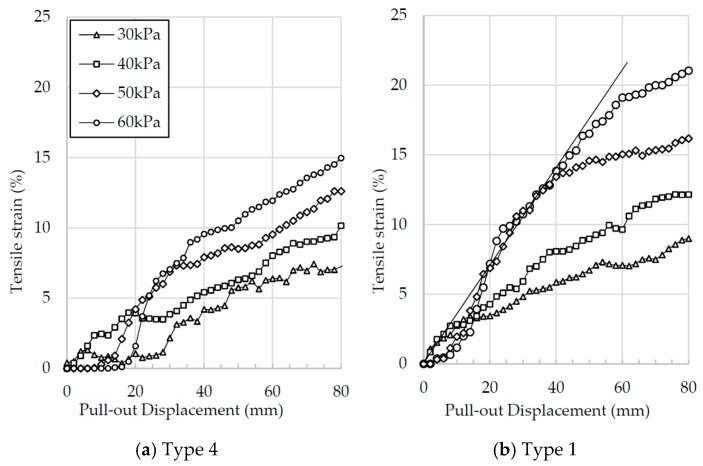
Average tensile strain at the middle of the tire strips plotted against the pull-out displacement for: (**a**) Type 4 tire strips; (**b**) Type 1 tire strips.

Figure 8 shows the average strains calculated at the middle of the tire strips for Types 1 and 4, for all the stresses tested. The results shown are similar to Figure 7: as the vertical stresses increase, the average mobilized tensile strains also increase, regardless of the tire strips type. Similarly, the effect of tire wear level affects the amount of strain mobilized by the tire strips: it is clear that the Type 1 tire strips mobilize higher strains than the Type 4, for all vertical stresses that were tested. This is similar to what was seen in Figure 4, where the Type 4 reached higher pull-out loads than the Type 1 tire strips.

Figure 8 shows that the average strains in the middle portion of the tire strips are much higher than the strains mobilized at the end of the strips. It also shows that the strains in this portion are mobilized much more quickly than the strains at the end portion, as shown in Figure 7. Figure 8 also shows that the levels of strain achieved in the middle part of the fibers are higher than the levels achieved at the end part; however, these are still much lower than the stresses seen at yielding on the direct tension tests. These different values of average strains (mobilized in the middle and end of the tire strips) imply that each part of the tire strip is mobilizing a different frictional strength, indicating that failure must be progressive, i.e., the elements nearer the pull-out clamp are reaching a failure strength earlier than the elements at the end of the strip. This seems to be confirmed by the rate of mobilization of tensile strains: as the pull-out displacement is applied, the strain mobilization reaches a maximum rate, indicated by the straight line starting at the origin of the graph. As the pull-out displacement progresses, it reaches a transition point where the rate reduces; this is likely to indicate that this portion of the tire strips have overcome the average peak strength, and are now mobilizing strengths towards critical values. The differences in slope between Figure 8a,b must be related to the higher stiffness of the Type 4 tire strips, as it requires fewer strains to reach a load capable of activating the failure mechanism.

### 3.4. Frictional Resistance and Relative Displacement

**Figure 9:**
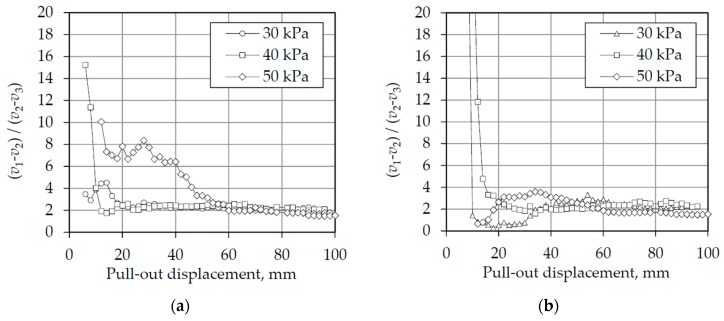
Ratio between the displacements measured in the middle and at the end of the tire strips for different normal stresses: (**a**) Type 1 tire strips; (**b**) Type 4 tire strips.

In order to better understand the distribution of deformations along the tire strips, the ratio between the displacements at the middle and at the end of the tire strips was calculated and plotted against the pull-out displacement in Figure 9 (for Types 1 and 4 tire strips). This shows that at the beginning of all the tests, this ratio was very large, indicating that the mobilization of strains occurs earlier at the middle part of the tire strips. As the pull-out displacement continues, more and more deformations are mobilized at the end part of the tire strip, reducing this ratio. As the tests progress, it seems clear that all converge to a ratio of 2, where the deformations in the middle of the fibre are twice as large as the deformations at the end of the tire strips. In this research, this value seems to be independent of the tire wear level and the stresses used, as both Types 1 and 4 reached the same ratio after a large deformation.

These results are consistent with [25], which concerns the distribution of tensile forces along a reinforcement embedded in soils and under a pull-out load. Similar findings were also observed on reinforcements embedded in sands (e.g., [26,27]).

## 4. Conclusions

This study presents an experimental investigation on the pull-out performance of tire-strips embedded on a sandy material, as an alternative to the use of more elaborated by-products of the tire industry. A series of pull-out tests were performed, where the load-displacement characteristics of sand reinforced with tire strips and uniaxial and biaxial geogrids was investigated and compared, for different vertical stresses. The results have shown that:

- The pull-out loads mobilized by the tire strips are two times higher than the pull-out loads mobilized by the uniaxial and biaxial geogrids, under the same testing conditions. However, the stiffness of the geogrid-sand system is larger than the stiffness of the tire strips-sand system.
- Tire strips reinforced sand fails by progressive failure, where each portion of the strip is mobilizing different strengths from the soil. At the point of failure, however, the strains mobilized on the tire strips were much lower than the strains measured when carrying out direct tensile tests on similar specimens.
- The interface strength properties that were calculated yielded values far higher than the strength properties of the sand. This indicates that the shearing interface being mobilized must be on the sand and away from the contact interface between sand and tire. Hence, more fundamental research is needed in order to understand how the rubber mobilizes larger strengths, and how to determine those properties.

## Figures and Tables

**Table 1:** Physical properties of Fujian Standard sand.

Parameter	Value
Effective size, *D*_10_ (mm)	0.25
Uniformity coefficient, *C*_u_	1.92
Coefficient of curvature, *C*_c_	1.40
Maximum dry density, *ρ*_max_ (g/cm^3^)	1.65
Minimum dry density, *ρ*_min_ (g/cm^3^)	1.33
Maximum void ratio, *e*_max_	0.99
Minimum void ratio, *e*_min_	0.61

**Table 2:** Engineering properties of the geogrids.

Properties	Uniaxial Geogrid	Biaxial Geogrid
Longitudinal tensile yield strength per meter (kN/m)	50.6	25.17
Transverse tensile yield strength per meter (kN/m)	-	25.2
Longitudinal elongation at yield (%)	15.0	10.5
Transverse elongation at yield (%)	-	10.3
Geometric size (mm × mm)	420 × 15 (each strip of geogrid)	40 × 35 (grid size)
Length (mm)	420	420
Tensile modulus under 2% strain levels (kN/m)	13.9	9.8
Tensile modulus under 5% strain levels (kN/m)	24.7	19.4

**Table 3:** Engineering properties of tire strips.

Properties	Type 1	Type 2	Type 3	Type 4
Longitudinal tensile yield strength of per meter (kN/m)	58.0	54.0	44.0	68.6
Longitudinal elongation at yield (%)	75.8	85.9	78.5	64.2
Size (mm × mm)	420 × 30	420 × 30	420 × 30	420 × 30
Tensile modulus under 2% strain levels (kN/m)	39.8	37.4	20.9	45.8
Tensile modulus under 5% strain levels (kN/m)	83.7	82.5	43.6	89.7

**Table 4:** Apparent Mohr-Coulomb shear strength parameters mobilized at the interface.

Reinforcing Material	Interface Friction Angle *φ* (degree)	Cohesion *c* (kPa)
Uniaxial geogrid	21.4	7.1
Biaxial geogrid	27.6	9.5
Type 1 tire	52.7	23.9
Type 2 tire	52.5	22.0
Type 3 tire	49.7	10.7
Type 4 tire	52.9	33.9
